# From Nano to Quantum: Ethics Through a Lens of Continuity

**DOI:** 10.1007/s11948-025-00557-w

**Published:** 2025-10-13

**Authors:** Clare Shelley-Egan, Eline De Jong

**Affiliations:** 1 Ethics and Philosophy of Technology Section, Department of Values, Technology and Innovation, Faculty of Technology, Policy and Management, Jaffalaan 5, Delft, 2628 BX The Netherlands; 2 Language and Computation, Institute of Physics, University of Amsterdam: Institute for Logic, QuSoft Stanford Center for Responsible Quantum Technology, Amsterdam, The Netherlands

**Keywords:** NEST-ethics, Nanoethics, Quantum ethics, Continuity, Quantum for good, Quantum technologies

## Abstract

A significant amount of scholarship and funding has been dedicated to ethical and social studies of new and emerging science and technology (NEST), from nanotechnology to synthetic biology, and Artificial Intelligence. Quantum technologies comprise the latest NEST attracting interest from scholarship in the social sciences and humanities. While there is a small community now emerging around broader discussion of quantum technologies in society, the concepts of ethics of quantum technologies and responsible innovation are still fluid. In this article, we argue that lessons from previous instances of NEST can offer important insights into the early stages of quantum technology discourse and development. In the embryonic stages of discourse around NEST, there is often an undue emphasis on the novelty of ethical issues, leading to speculation and misplaced resources and energy. Using *a lens of continuity*, we revisit experiences and lessons from nanotechnology discourse. Zooming in on key characteristics of the nanoethics discourse, we use these features as analytical tools with which to assess and analyse emerging discourse around quantum technologies. We point to continuities between nano and quantum discourse, including the focus on ‘responsible’ or ‘good’ technology; the intensification of ethical issues brought about by enabling technologies; the limitations and risks of speculative ethics; the effects of ambivalence on the framing of ethics; and the importance of paying attention to the present. These issues are taken forward to avoid ‘reinventing the wheel’ and to offer guidance in shaping the ethics discourse around quantum technologies into a more focused and effective debate.

## Introduction

A significant amount of scholarship and funding has been dedicated to ethical and social studies of new and emerging science and technology (NEST), from nanotechnology to synthetic biology, and more, recently, Artificial Intelligence (AI). Quantum technologies comprise the latest NEST, attracting interest from scholarship in the social sciences and humanities. This family of technologies leverages the principles of quantum mechanics in the development of advanced technological capabilities, and promises to enable applications ranging from enhanced medical imaging and mine detection (quantum sensing), to improved climate modelling and accelerated drug discovery (quantum computing), and inherently secure communication (quantum networks).

Anticipating these applications of the future second generation of quantum technologies, discourse around the ethics and, more broadly, the responsible innovation of quantum technologies, is slowly gaining momentum. Unlike other areas of new and emerging science and technology (NEST) like nanotechnology, a broad constellation of actors has not yet emerged around the discourse, in terms of developments such as civil society organisation (CSO) engagement, public dialogue exercises, contribution from the social sciences and humanities, and so on (Vermaas, 2017; Coenen & Grunwald, 2017). Rather, discourse around quantum technologies and society has remained within the remit of governmental and corporate actors (Vermaas, 2017). While there is a small community now emerging around broader discussion of quantum technologies in society (Vermaas, 2017; Ten Holter et al., 2021; Coenen et al., 2022; Hoofnagle & Garfinkel, 2022; De Jong, 2022; Seskir et al., 2023; Kop et al. 2024; OECD, 2024), the concepts of ethics of quantum technologies and responsible innovation are still fluid and emerging. This article aims to contribute to shaping this emerging field of ethics by drawing lessons from previous NEST discourses.

In the embryonic stages of discourse around NEST, there is often an undue emphasis on the novelty of ethical issues (cf. Nordmann, 2007), potentially leading to speculation and misplaced resources and energy. Moreover, a focus on novelty can lead to a backgrounding of strong continuities with previous NEST and their associated ethical discourse. Ethicists and social scientists involved in the study of NEST are not immune to hype, with a myriad incentives to emphasise novelty (cf. Seifert & Fautz, 2021), not least the mobilisation of funding and resources. In this article, we argue that lessons from previous instances of NEST can offer important insights into the early stages of quantum technology discourse and development.

Using *a lens of continuity*, we revisit experiences and lessons from nanotechnology discourse. The ‘nano’ discourse offers a valuable case for historical lessons for quantum ethics, not only because it is a ‘prototype’ of NEST and thus offers a paradigmatic case for ethics of NEST or ‘NEST-ethics’, but also because the technologies and their associated impacts are of a comparable character: both ‘nano’ and ‘quantum’ can be considered “system technologies,” representing a family of technologies with inherently open-ended applications, leading to a wide array of potential impacts. Furthermore, it can be said that quantum technology development and related discourse is roughly at the same stage of development as was nanotechnology in the early to late 2000s. By exploring the nanotechnology discourse, we offer insights that can help the burgeoning community to take stock of experiences and past achievements, rather than ‘reinventing the wheel’. While our focus in this article is on the risks of overemphasising novelty^1^ (the ‘novelty trap’) and, by extension, on the importance of recognising continuities, we acknowledge that there is also a risk of dismissing developments as mere reiterations of past concerns (the ‘nothing-new trap’). Our search for continuities between the emerging quantum ethics discourse and the nanoethics discourse does not preclude the possibility of genuine discontinuities. However, since the current discourse on quantum technologies tends to emphasise their novelty—focusing on questions like “how is quantum technology *different* or *new* compared to other technologies?” (e.g. Possati, 2023)—we argue that continuities risk being overlooked and therefore deserve explicit attention.

Furthermore, we seek to advance a modest and resourceful approach to the development of the ethics of quantum technologies in which we – as ethicists and social scientists – are mindful of our responsibility. We understand responsibility here as our responsibility both to assess potential consequences and impacts, along with reflecting on ethical discourse on a more abstract level. We pursue our approach by mobilising past research in the ethics and responsible development of nanotechnology in which the first author participated, namely the DEEPEN (Deepening Ethical Engagement and Participation in Emerging Nanotechnologies) project. DEEPEN was a three-year project which ran from 2006 to 2009 and was funded by the European Commission’s Framework Programme 6. The project sought to integrate understanding of the ethical challenges posed by emerging nanotechnologies in ‘real world’ situations, along with their implications for publics, for governance and for scientific practice. The reflections that follow aim to draw lessons from this experience for how to responsibly engage with the opening stages of discourse around quantum technology development. Both authors are currently involved in projects that seek to address the broader ethical and societal dimensions of quantum technology development. The first author is a researcher in the ‘Quantum and Society’ Action Line of the Centre for Quantum and Society (CQS) in the Netherlands. CQS is part of the Dutch quantum ecosystem Quantum Delta NL and is funded by the National Growth Fund.^2^ The Centre comprises both a knowledge and co-creation centre focused on advancing the beneficial impact of quantum technologies on society and facilitates ELSA research and science communication. The second author is a researcher in a project about the impact of large-scale quantum computers on cybersecurity^3^ and is a research fellow at the Stanford Center for Responsible Technology. Thus, while we base our analysis and discussion of ethics of quantum technology development on the extant literature, we also draw on our active engagements in the world of quantum development. We are ‘moving about’ (cf. Rip & Robinson, 2013) in this emerging field – through our own research projects and collaborations, visiting sites of quantum R&D, participating in conferences and other interdisciplinary meetings, and closely following coordinating activities, such as those by the Open Quantum Institute, the European Quantum Flagship, the World Economic Forum, the OECD, along with national strategies and strategic research agendas, in which developments are being shaped. This form of situated engagement allows us to capture what is happening, to acquire rich knowledge about dynamics and contexts and to generate grounded diagnoses about what is happening and could happen.

We begin in Sect. 2 by defining a thesis of continuity, based on the work of Joly (2015) and on key characteristics shared by NEST (cf. Grunwald, 2016) that are relevant for ethical discourse. In Sect. 3, we outline and describe key features of the nanoethics discourse, which serve as the analytical frame for examining the emerging quantum ethics discourse. In Sect. 4, we describe the contemporary (ethical) discourse on quantum and mobilise our analytic frame to analyse it. In Sect. 5, we conclude by emphasising the importance of adopting a heuristic of continuity, using past experiences to advance the current ethics discourse around quantum technologies.

## Mitigating a Tyranny of Novelty: A Thesis of Continuity

### Thesis of Continuity

We begin with an assertion that we should avoid ‘reinventing the wheel’ when considering the potential embedding of a new technology in society. We contend that, to a considerable degree, quantum ethics will be NEST ethics—just like nanoethics also raised ethical questions that applied to NEST more generally. Nonetheless, some adaptations may have to be made to the specific characteristics of and dynamics around quantum technologies (Ferrari, 2010).

Offering a paradigmatic case of NEST- ethics, we contend that the discourse on nanoethics can offer important insights for an effective quantum ethics. Lessons learned from the experience of TA NanoNed^4^ are particularly instructive here. Rip and Van Lente (2013) observe the following regarding Technology Assessment and ELSA (Ethical, Legal and Social Aspects) studies:

TA and ELSA studies have to be defined in relation to the actual situation of, and imminent challenges for, nanotechnology in society. There is no context and history independent way to do TA and ELSA, they have to reinvent themselves again and again (p. 14).

While there may be no “context and history independent way to do TA and ELSA”, this does not automatically imply a novel approach to studying the embedding of quantum technologies in society. Indeed, we seek to move away from the ‘novelty trap’, described by Rayner (2004):

The emerging controversy over nanotechnology is redolent of the pattern of past disputes over new technological fields (…). In each case, ambitious claims for revolutionary innovation are met by sceptical concerns about unintended consequences and new risks, to which advocates respond with reassurances of continuity with past experience (p. 349).

The novelty trap points to a lack of social or institutional learning “that can effectively inform the process of introducing new fields of technological endeavour” (Rayner, 2004, p. 351). Joly (2015) elaborates on the novelty trap with reference to the ‘tyranny of novelty’; an “obsession with novelty”, he suggests, may lead to the adoption of “a narrow frame for the analysis of emerging technologies” (Joly, 2015, p.14). This narrow frame is founded on limitations identified by Joly of anticipatory governance approaches to emerging technologies. First, there is a lack of attention to the past and the present in analyses of the future. A new technology does not emerge out of thin air; socio-technical imaginaries are based on past and present structures and discourses, which, in turn, feed into our visions and interpretations in uncertain contexts. Second, issues of power tend to be neglected in distributed governance discourse – such as responsible innovation discourse – such that power relations and resource distribution are rendered invisible, along with the various forces that inform research agendas.

An alternative to the tyranny of novelty is what Joly (2015) terms a ‘heuristic of continuity’. The heuristic of continuity is designed to overcome the tyranny of novelty and a focus on uncertainty to what we *do* know. It implies drawing lessons from the history of technology while highlighting the specific features of contemporary emerging technologies. Such lessons centre on insights, e.g. regarding recurring discursive regimes linked with general understanding of the role of technologies in society (for instance, a master narrative of progress vs. risk society), along with “(…) schemes and sets of routines that naturalize the use of technological innovations (…)” (p. 17). Insights also rest on past technoscientific investments, and actors’ positions and interests with respect to the framing of ‘novelty’. The regulation of technologies comprises another important historical insight. Technologies are regulated through “institutions and instruments which have their own existence” (p. 17). While new technologies carry their own specific hazards, patterns exist in how they are regulated, e.g. a narrow framing of risk which emphasises environmental, health and safety aspects, while leaving out ethical, societal and broader dimensions, and political relations. Moreover, there are patterns in the role of risk analysis and risk management in how new technologies are constructed as being acceptable.

### Defining NEST (ethics)

Both nanotechnology and quantum technology share common characteristics of NEST, which are of significance for ethical discourse (Grunwald, 2016). We discuss key characteristics here. First, NEST developments blur the classical boundary between technology and science—with technology still being in a ‘scientific’ phase^5^—leading to new challenges for the assignment of responsibility and a specific NEST-ethics. Second, NEST developments provide *enabling technologies*, meaning that they can be taken up in a variety of applications in widely varying fields, with the implication that potential impacts will be diverse and anything but unambiguous. Third, the difficulty of assessing techno-futures of NEST (due to the combination of novelty and ambiguity) has led to certain *patterns of communication* that fall back on hype, hope, and fear, attracting a significant degree of attention from public and political spheres, and potentially impacting opinion forming and decision-making. An additional issue is that of *governance*; new and emerging science and technologies pose a challenge for governance with respect to uncertainties about their functionalities and risks (Kearnes et al., 2006; Rip, 2010). The notion of centralised governance and its illusion of control is undermined by NEST (Rip, 2019); instead, modulation of ongoing dynamics is possible.

Ethical discourses emerge from NEST. Since NEST share some key characteristics, the ethical issues that they raise also exhibit some similarity. Zooming in on the patterns in NEST-ethics discourses, Swierstra and Rip (2007) identify some recurring meta-ethical issues. Part of the argumentation about NEST occurs at a meta-ethical level “about our background understanding of the issues and how to approach them, rather than about substantial questions about good action and the good life” (p. 7). We summarise these recurring meta-ethical issues as described by Swierstra and Rip in the following. First, a presupposition of NEST-ethics that the development of a new technology can be influenced leads not only to discussions of desirability and feasibility but also to long-standing discussion about technological determinism, with patterns of moral argumentation divided, and dependent on the audience and the situation. Thus, for example, proponents might advocate a determinist approach but be more circumspect about technology development in private, while, at the same time, actors will position themselves differently with respect to the new technology, according to whether they are proponents (e.g. insiders) or critics (e.g. publics or representatives of civil society). Second, past experiences with new technology are drawn upon to argue both for precaution *and* to emphasise ‘business as usual’: both novelty and the ‘nothing new’ perspective function as recurring topoi in NEST ethics discussions. A third main pattern of meta-ethical argumentation centres on the possibility that NEST may change our moral and ethical considerations, with such change considered alternatively as something inevitable or as a threat. These patterns of meta-ethical argumentation can be used as heuristic tools with which to map and elaborate how such arguments played out in the nanoethics discourse. We implicitly make use of these tools in the following section.

## Mobilising the Past: Key Features of the Nanoethics Discourse

In this section, we examine the emergence and key characteristics of the nanoethics discourse from the early 2000s to the early 2010s, drawing in particular on insights from the DEEPEN project. Since our aim is to analyse the present discourse on quantum technologies through the lens of past debates, a thorough engagement with the nanoethics discourse is necessary. This extensive discussion is essential to identify patterns and continuities that inform our analysis of quantum ethics. Moreover, to fully ‘mobilise the past,’ we adopt a broad analytical scope, which means that the aspects we highlight operate at different analytic levels—some addressing specific trends in how nano discourse was framed, with others reflecting broader structural tendencies in how ethical discussions are conducted. This allows for the development of a ‘multifarious lens’ for our later analysis of the quantum ethics discourse.

### Genealogy of the Nanoethics Discourse

Before reflecting on the defining aspects of the nanoethics discourse, we first briefly examine its emergence, as this provides important background for the remainder of our discussion. In the case of nanotechnology, the NEST characteristics described in the previous section surfaced in the felt need to “get it right the first time”, given concerns expressed about possible negative impacts of the new and emerging technology, and concerns from promoters of nanotechnology that lessons from biotechnology and agricultural genetically modified foods and crops about public acceptance should be taken up from the very outset (Kearnes et al., 2006). Experimental approaches to governance were experimented with by a variety of actors, from governments and companies to social sciences and humanities (SSH) scholars. Indeed, the domain of nanotechnology was the incubator for a multiplicity of efforts in ‘responsible development’ (cf. Rip, 2014; Grunwald, 2014).

The field of ‘nanoethics’ was a key part of the responsible development endeavour. The development of the nanoethics agenda comprised various strands and emphases. In the early 2000s, the approach of bioethics was taken up in the ethics of nanotechnology, e.g. in the compilation of inventories of ethical issues (Ebbesen et al., 2006; Lewenstein, 2006). There was very little reflection on the specific characteristics of nanotechnology, with the effect that ethical debate on nanotechnology was reduced to rote checklists of basic concerns. Consequentialist frameworks emphasised issues linked to the risks of nanoparticles such that ethical concerns were framed in terms of the willingness or lack thereof to accept these risks (Ferrari, 2010). In the run-up to the 2010s, increased acknowledgment of the narrowness of the consequentialist framework took place; there was a call for a heightened focus on the motives and scope of technological development and reflection on issues such as sustainability and responsibility as a means of dealing with the uncertainty of nanotechnology (UNESCO, 2006; European Commission, 2008). Thus the nanoethics discourse developed from early concerns about public acceptance, progressed through bioethics, and finally landed on more nuanced approaches. In the next section, we will highlight some of the features that defined the discourse.

### ‘Responsible Development’ and Nanoethics: A Conversational Mode

The notion of ‘responsible development’ emerged as a key framework for governance with which to orient the development of nanoscience and nanotechnology. The incorporation of the notion of ‘responsibility’ into research and innovation policy and its subsequent “quick career” (cf. Grunwald, 2014) can be viewed as having a dual role (cf. Shelley-Egan, 2011). On the one hand, responsible development was a response, primarily at policy level, to the novelty of nanotechnology and an attempt to put responsibility on the agenda. On the other hand, the call was something that impinged on the *de facto* roles and responsibilities of actors in the nano-world. Responsible development activities encompassed soft law mechanisms, e.g. the development of codes of conduct, dialogue initiatives and research on the Ethical, Legal and Societal Aspects (ELSA) of the technology. A key criticism of the responsible development ‘movement’ made by the DEEPEN consortium bears repeating here; this is what is termed the ‘conversational mode’ in which the responsible development of nanotechnology was carried out. In essence, the project characterised this mode as a sort of conversation about nanotechnology and its responsible development to which stakeholders and citizens were invited to contribute, along with nano-researchers and policymakers. This is elaborated as follows:

It is a conversation precisely in the sense that everyone can join in and say their piece: it is an open-ended, accommodating, process that incorporates opinions that vary considerably in topicality and scope. Since no participant in a conversation is thought to speak from a position of self-interest and since the conversation does not culminate in a decision that will cost some and benefit others, participants need not prevail over each other but can afford to listen as various concerns are raised. (Nordmann & Macnaghten, 2010, p. 135).

In sum, then, this conversational mode downplayed stakes and smoothed out conflictual situations. The challenge for ethics identified by the project, is that ethics discourse becomes part of this conversational mode. ‘Ethics’ becomes a kind of *lingua franca* that functions to bring stakeholders together and make them aware of each other but does not go much further than that. This ‘ethicalisation’ – or translation of technological development into questions of ethics (Davies et al., 2009) – has important consequences. On the ”shared platform of ethics” in which an open-ended sharing of ethical concerns takes place, ‘(un)ethical’ is another word for what is ‘good’ or ‘bad’ (Ferrari & Nordmann, 2010, p. 172). The political character of ethical issues is dampened; e.g. a conversational mode limits the discovery of “grave ethical concerns” that might comprise a barrier to continued development of nanotechnologies.

### An Emphasis on Novelty

A key element of ethical discourse on nanotechnologies took place through what has been termed a ‘novel ethics’ frame, that is an ethics approach based on the novelty of issues (cf. Moor & Weckert, 2004; Nordmann, 2007; Rehmann-Sutter & Scully, 2010; Ferrari, 2010). In the early 2000s, debate abounded regarding the extent to which nanotechnologies posed novel ethical issues and warranted a special ‘nanoethics’. On the one hand, there was a camp in favour of a dedicated field of nanoethics, with one argument centering on the environmental, health and safety issues relating to nanomaterials that required dedicated attention (Allhoff & Lin, 2006). On the other hand, those against the delineation of a new ‘nanoethics’ field argued that the novelty of issues presented a poor “indicator of their relevance and urgency for ethical reflection” (Rehmann-Sutter & Scully, 2010, p. 242). Others in this camp positioned ”old questions” as having the potential to be “good questions” (Rehmann-Sutter & Scully, 2010, p.243).

The emphasis on novelty is a recurring theme in the nanoethics discourse and may implicitly shape some of the features we discuss next. However, we wish to treat it as a distinct aspect, as this allows us to examine the discourse from different angles, rather than imposing a single interpretive lens.

### Nanotechnologies Intensifying Ongoing Trends

Furthermore, old ethical questions (e.g. about desirability, privacy, justice, etc.) were framed as requiring careful (re)consideration in light of the status of nanotechnologies as enabling technologies (Swierstra & Rip, 2007):

By making existing technologies smaller and faster, well-known ethical issues, say privacy and new ICT, or point-of-care diagnostics and professional-medical responsibilities, can become more pressing, but not necessarily different in kind (Swierstra & Rip, 2007, p. 3).

Nanotechnologies were described as not necessarily introducing radically discontinuous changes but *intensifying ongoing trends*, e.g. such as the ‘medicalisation’ of society, with medical nanotechnology allowing for improved monitoring and measuring (Ferrari & Nordmann, 2010). This intensification, Ferrari and Nordmann suggest, points to a challenge to “(…) look for the motives and ideas that support these trends, and (…) to ask what happens if nanotechnologies push them even further and perhaps to their limit” (p. 175). This challenge reflects a move away from a search for novelty to one that is grounded in current logics and trends.

### Minding the Gap between Ethics and (nano)technology

The speculative nature of nanoethical debate became a major current in the discussion. Nordmann (2007) argued that speculative nanoethics wastes ethical resources that could be best oriented towards developments that are taking place as opposed to those that are still hypothetical and speculative. Speculative ethics diverts attention from ‘less exciting’ but no less important developments. In their ‘Mind the gap revisited’ commentary, Nordmann and Rip (2009) represented their concern about speculative ethics in terms of a gap in which ethics leaps ahead while current science is left behind. They recommended two strategies for closing the gap in the area of nanotechnology. The first strategy emphasises a need for discussion about the ‘quality of promises’ and the making of ‘distinctions’, as follows:

Just as everything that is physically possible is not always technically feasible, everything that can benefit an individual will not automatically benefit the whole of society. Distinctions need to be made that cut down to size the supposedly unlimited potential of nanotechnology (p. 274).

Given the speculative nature of expectations surrounding emerging technologies, Nordmann and Rip call for a ‘reality check’ prior to engaging in ethical discussions. This, they argue, is essential in preventing (nano)ethics from uncritically following technological hypes. Scrutinising the credibility of such promises is intended to support a more rational and substantive ethical debate.

The second strategy centres on the need for ethicists to help differentiate between ‘the’ technology as a whole, and concrete applications of the technology. For the case of nanotechnology, this difference was evident in the “extremely general ideas (…) associated with nanotechnology in the singular (…) and the various challenges (…) presented by nanotechnologies in the plural” (Nordmann & Rip, 2009, p. 274). Thus, in the case of nanotechnologies, antibacterial surfaces surfaced different ethical questions than that of the future miniaturisation of semiconductors.

Without denying the risks of ethics becoming ‘too speculative’, scholars such as Roache (2008), Grunwald (2010), Selin (2014) and Urueña (2021, 2023) have argued that ethical debates should extend beyond existing technologies to consider potential future developments. In that sense, ‘speculation’ is inherently part of a forward-looking ethics. This is also acknowledged by Nordmann and Rip (2009). Consequently, ethics in an anticipatory mode must engage with future possibilities—yet the challenge lies in doing so thoughtfully, while mitigating the risks associated with speculation.

### From Speculative Ethics to Standing in the Present

The critical discussion about speculative ethics came with a call to shift focus to motives and dynamics around technology development. An important part of this discussion is the idea of a “re-appropriation of the present” (Ferrari, 2010). Based on a critical analysis of nanoethical discourse at the time, Ferrari called for approaches that focus effort on the present and on a “critical confrontation” with past technological experiences (p.46). ‘Understanding the importance of the present’ underlines reflection on forms of governance and the social context in which NEST are being developed, while ‘reclaiming the past’ implies learning from past experiences, not only from errors but also to allow for a more “detached view of social and political dynamics” (ibid., p. 46).

Elsewhere, Ferrari and Nordmann (2010) elaborated on what a focus on the present would mean for nanoethics:

Instead of gazing only at what might come out of nanotechnological research, nanoethics needs to consider and evaluate what, concretely, goes into nanotechnological development. That is, it needs to look at funding priorities, research programs, technological visions, long-term trends like medicalization or ethicalization, old and new hopes, abstract and concrete fears (p. 175).

This ‘re-appropriation of the present’ represents what Grunwald (2014, 2017) terms the most upstream stage of NEST-engagement. This upstream stage emphasises the attribution of meaning, as regards the content of the techno-future and the actors who are doing the assigning. Meaning can encompass “(…) what will be viewed as chance or risk, what will be included and excluded in debate, and which kind of innovation and application areas will be emphasised” (Grunwald, 2017, p. 101). Ethical discussion and debate about responsibility emerge from the intertwining of scientific expectations or projects and their possible social meanings. Depending on which meaning prevails, the NEST is assigned to a specific discussion, put in a different context, and attributed different meanings (Grunwald, 2014, p. 276). Scholars like Grunwald suggest shifting the focus of ethical evaluation of imagined outcomes to the broader visions that underpin these speculations (Grunwald, 2014). Building on the call to make the attribution of meaning an object of ethical reflection, the nano discourse, therefore, prompted a call for “vision assessment” (Grunwald, 2014).

### Ambivalence of Promising Technology

Ambivalence became visible in the early days of nanotechnology discourse. Indeed, ambivalence is a characteristic of the governance of new technosciences; ambivalence can be understood with respect to a dual narrative about a new technology, as described by Felt et al. (2007):

The technology is *brand new* (and will create a new society through genetic modification or offer nano-implants for human enhancement) when technological elites speak to investors, policy makers or patent offices, and to publics to be enrolled in the new venture. But at the same time technology is *nothing unusual* (we have been modifying genetic make-up of organisms all the time, nanotechnology is just about making things smaller and faster) when actual or anticipated concerns have to be assuaged (p. 26; our italics).

This aligns with the recurrent pattern in NEST ethics of stressing either novelty or ‘business as usual’ (Swierstra & Rip, 2006).

Discourse on the responsible development of nanotechnologies began with the promise of the technology and then quickly narrowed down to specific ambivalences of the technology, for example, the idea that “size matters” (Shelley-Egan, 2010). “Size matters”, on the one hand, due to the interesting phenomena and novel effects that takes place at the nanoscale. On the other hand, “size matters” with respect to the potential toxicity of nanoparticles. This ambivalence created and fuelled discussion – and contestation – between supporters and opponents of the technology (Swierstra & Rip, 2007; Shelley-Egan, 2010). The ‘ethics of promising technology’, or “Thou shalt not exaggerate without reason”, as described by Swierstra and Rip (2006) was part of this ambivalence. The reason to exaggerate for nanotechnology rested on the need to mobilise and attain resources in a resource-competitive environment: “One has to claim more than is reasonable, in order to be able to realize what is actually a reasonable claim” (p. 18). There was broad recognition of this issue in the nano-world. Moreover, as observed by Swierstra and Rip (2006), it also leads to an ambivalence in the sense that different actor strategies are possible: “Be willing to inflate expectations, and hope disappointment will not run high. Or be modest to avoid a backlash” (p. 18).

## Ethical Engagement with Quantum Technologies

In this section, we draw on insights from the nanoethics debate to structure and guide our analysis of the emerging ethical discourse on quantum technologies. Given that the quantum ethics debate is still in its early, exploratory phase, the available material for analysis remains limited, making our assessment necessarily preliminary. Nonetheless, engaging in this early-stage reflection fosters greater reflexivity within the field amongst ethicists and responsible innovation scholars and, by extension, contributes to the quality of the evolving discourse.

### The Emergence of the Quantum (ethics) Discourse

Quantum technology is not new – it has been used already in semiconductors, lasers and MRI scanners and is the technological foundation for computers, smartphones and the Internet (Rathenau Institute, 2023). Whereas this first generation of quantum-based technologies relies on quantum principles without actively manipulating quantum properties, scientists and engineers are now working on a second generation that directly harnesses and manipulates quantum properties—such as superposition, interference, and entanglement. This could enable applications that are “faster, more accurate, smaller, cheaper, or in other ways better, in some cases by order of magnitude” (Inglesant et al., 2018, p. 11). The most emblematic example of this new wave is the envisioned quantum computer, which aims to perform certain computations exponentially faster than the most powerful classical computers—though this advantage is expected to be significant only for specific types of problems.

At the forefront of quantum technology development are major technology companies such as IBM, Google, and Microsoft, alongside specialised firms like IonQ, Rigetti, and D-Wave. Academic research institutions remain central to progress in the field, with universities such as MIT, Oxford, Delft University of Technology, and the University of Science and Technology of China leading breakthroughs in quantum computing, quantum communication, and quantum sensing. Many of these academic efforts are supported through government-backed initiatives, including the US National Quantum Initiative, the European Quantum Flagship programme, and China’s substantial state investments. In fact, global competition among governments has driven the launch of various national quantum strategies— at least 32 countries had a national quantum initiative or strategy as of August 2023 (GESDA, 2023)—with China and the US being the largest national investors. This effort is likely to be further boosted by UNESCO’s 2025 International Year of Quantum Science and Technology.

While quantum developers and supporters herald the possibilities of quantum for society – even being described as a potential “game-changer” in many social and economic sectors (Quantum Delta NL, 2019, p. 37) – the technology is still largely in the development phase, with some first applications breaking through. Applications of quantum technology tend to be differentiated along three different areas, namely, quantum sensing, quantum computing and quantum communication.

Quantum sensing, which allows measurements at the atomic level, has demonstrated technical viability and is already being explored for applications in medical imaging, quality control, navigation, and detection of underground objects (Stray et al., 2022; Aslam et al., 2023; Kantsepolsky et al., 2023; Q.ANT (2024, April 9). In contrast, quantum computing and quantum communication remain in early developmental stages. Despite steady progress over recent decades, breakthroughs have yet to translate into commercially viable, large-scale applications, and research remains largely fundamental. Competing hardware approaches for quantum computing—such as trapped ions versus superconducting qubits—are still being explored, with no clear frontrunner. If realised, full-fledged quantum computers could revolutionise fields that rely on computational power, such as optimisation, complex system modelling, artificial intelligence, and drug discovery, while also posing risks to current cryptographic methods (Hoofnagle & Garfinkel, 2022; Preskill, 2023; Santagati, 2024; Groenland, 2025). Quantum communication, and eventually a “quantum internet,” aim to enable inherently secure information transfer, with potential applications in sectors handling sensitive data, such as healthcare, finance, and national security (Zhang et al., 2022; Pan et al., 2024; Gupta et al., 2024; Sutradhar et al., 2024).

The potential applications of quantum technologies have sparked calls in academic literature for responsible innovation and ethical reflection (Coenen & Grunwald, 2017; Ten Holter et al., 2023; Possati, 2023; Gasser, Kop, & De Jong, 2024; Kop et al., 2024;). These calls are echoed in policy documents, research initiatives, white papers, workshops and conferences on responsible quantum technology. Across these efforts—whether in academic discourse or the policymaking sphere —the ethical discourse remains largely centred on *agenda-setting*, emphasising that quantum technologies warrant ethical engagement, and *exploratory* in nature, working to map out the ‘loci’ of potential ethical implications. Instead of a mature ethical debate that critically examines a concrete technology, the ethical discourse surrounding quantum technologies remains ‘fluid.’ This fluidity arises from the profound uncertainties surrounding not only *when* but *how* these technologies will ultimately materialise. Consequently, the ethical discourse is still focused more on debating the right approach to ethics at this stage and identifying key issues, rather than engaging in structured ethical analyses.

As compared to the early days of nanotechnology when risk and environmental, health and safety (EHS) issues were high on the agenda, current discourse around quantum technology development rests largely on its central role in national and economic competitiveness. There are two points relevant to ethics that can be made following this observation. First, it is governmental and corporate actors who currently play a key role in mapping economic prospects and mobilising resources, visions and strategic agendas, with a wider debate on quantum technologies between researchers, governments, industry, ethicists and social scientists and stakeholders in society not taking place (Vermaas, 2017). While this situation has evolved since 2017, with the establishment of two national centres dedicated to ELSA and responsible innovation of quantum technologies^6^, the same flurry of activity around quantum and SSH as occurred for nanotechnology (cf. Seifert & Fautz, 2021) has not (yet) come about. This may have implications for the early ethical ‘frame’ in which quantum development takes place, with corporate and other powerful interests potentially dominating such framing.

Second, while appeals to the importance of NEST in delivering on economic and national competitiveness are not new, quantum technology development does differ, with “(…) a more pervasive sense of international competition and conflict that spurs innovators and investors to action” (Coenen et al., 2022, p.1). This difference is further underlined by the “sociomaterial specificities” (Inglesant et al., 2021, p. 1366.) attached to quantum technologies such as, e.g. supply chains for critical raw materials. Given this specific context of technological development, one could say that the ethical stakes are more closely intertwined with broader societal and political trends and priorities, thus necessitating ethical discourse with a broader scope, addressing issues of politics and power.

### Continuities Between the Nano and Quantum Discourse: Taking Advantage of the Past

In the following, we apply insights from the nanoethics discourse described above to the case of quantum technologies. While we discuss the items separately, we do so for stylistic and communication reasons, as there are clearly overlaps and synergistic elements across the listed items.

### ‘Quantum for Good’

‘Quantum for good’ can be seen as a signifier for a particular direction of technology development. The notion can be said to fall under the umbrella of ‘Tech for Good’ as a concept that underlines the development or implementation of technology for the common good (Powell et al., 2022). ‘Quantum for good’ also echoes the notion of the ‘responsible development’ of nanotechnology in the sense that both labels emphasise a desirable world that is possible through technology development.^7^ In addition, both ‘quantum for good’ and ‘responsible development’ of nanotechnology highlight the importance of the context of application of the respective technologies in our societies.

‘Quantum for good’ has emerged from different places. Within the Dutch context, it has emerged top-down from the Board of Quantum Delta NL (Vermaas & Mans, 2024). The World Economic Forum (2022) stresses the importance of guiding the governance of quantum computing for the ‘benefit of humanity’. The notion of ‘public good’ and importance of public values with respect to quantum technology development has also been highlighted by commentators (Roberson et al., 2021; Rathenau Institute, 2023). The Rathenau Institute, for example suggests that while “(…) the new quantum technology has not permeated very far into society” (p.15), it is nonetheless worth considering societal issues through the lens of public values such as cybersecurity, justice, strategic autonomy, and sustainability, to name some of the issues. Specific use cases of quantum, e.g. quantum computing, are also being mobilised for the ‘good’. For example, the Geneva Science and Diplomacy Anticipator (GESDA) emphasises quantum computing in the service of addressing the Sustainable Development Goals (SDGs) (GESDA, 2023).

In quantum for good, the technology itself is put front and centre, and ethics and responsible innovation approaches are set to be mobilised to attain the goal of quantum for good, however it comes to be defined. Thus, the question as to whether quantum is a technology worth considering is answered from the very outset – we should pursue quantum for the right reasons and with the right purposes in mind. In this sense, ‘quantum for good’ is an ‘ethical’ qualifier in the sense that it indicates what is good (and should be done) (cf. Swierstra & Rip, 2007). It advances a particular ethical frame for technology development. Interestingly, in the absence of a normative label before ‘quantum’ such as ‘responsible’ or ‘ethical’, the idea that ‘for good’ also points to what is bad (and should not be done) is perhaps less present. Overall, the use of quantum for good can be viewed as incorporating ethics, not necessarily an ethics of quantum but an ethics of progress—and, to a lesser degree, an ethics of negative impacts through new technology (Swierstra & Rip, 2007).

Why and how has quantum for good gained traction as a guiding trajectory for quantum technology development? This question includes both conceptual and empirical aspects. As regards the latter, an emphasis on quantum for good can be positioned as part of the continuing trend of actively anticipating the responsible societal embedding of new technologies. Indeed, an interesting emphasis in the discourse on quantum technology development is on ‘meaningful’ development (cf. Coenen et al., 2022). Coenen et al. (2022), emphasise ‘meaningful’ quantum technologies as technologies that “engage with a greater variety of societal needs, hopes, and concerns, steering the development of quantum technologies toward applications that are meaningful not only for industry but also for society” (p. 5). Given the corporate and governmental dominance of quantum technology development, attention to their use by such entities, along with related concentrations of power, warrant careful attention in ethical discussion. On the other hand, there is also an element of impression management and a need to press ahead with development. Quantum for good requires operationalisation, supported by concrete, dedicated activities. Thus, one should take the articulation of ambitions such as quantum for good seriously but also look at *who* is voicing the ambition and ethos of quantum for good. It is important to be attuned to the possibility of a conversational mode also emerging for quantum development and quantum ethics such that stakes are downplayed and the political character of quantum for good is diluted in ethical discussions.

### Radical Novelty or Radical Intensification?

Quantum technologies are often presented as a class of technologies that are ‘radically different’ from all that have come before. This perception stems from the fundamental contrast between quantum physics and classical physics. Given that all current and past technologies are based on classical physics, quantum technologies do indeed appear to introduce a fundamentally new approach to technology design by harnessing the principles of quantum mechanics. This perceived novelty forms the basis for an argument that this new approach will unlock a range of unprecedented capabilities. Consequently, this promise has not only sparked calls for ethical scrutiny of these new capabilities, but also for a dedicated ‘quantum ethics’^8^ (Perrier, 2021; Possati, 2023). Perrier (2021) argues that the “unique features of quantum information processing may give rise to unique ethical concerns” (p. 6). ‘Quantum ethics’ focuses on navigating these concerns. Possati (2023) goes further and argues that quantum technologies require a new approach to ethics, stating that “compared to other forms of technology ethics, such as AI ethics, the ethics of QT is more complex and delicate” (p. 1), and that quantum computing “poses new ethical issues that require new conceptual tools and methods.” (p. 4) The idea is thus that the specific nature of quantum technologies requires a quantum-specific approach to ethics.

To avoid being captured by the allure of novelty and to benefit from past experiences, it is crucial to draw parallels with the discourse surrounding nanotechnology. As discussed in the previous section, the nano discourse similarly called for a distinct “nanoethics.” However, this call faced criticism as the novelty of the issues was not considered a valid indicator of the relevance and urgency for ethical reflection. Quantum technologies reinforce this critique, as it remains unclear how their novelty translates into genuinely new ethical issues. Rather than introducing entirely new ethical questions, quantum technologies are more likely to intensify well-known ethical challenges. This potential for intensification was highlighted in the nano debate, where it was argued that the amplification of existing trends deserved more attention than the speculative implications of future applications. The current quantum discourse does acknowledge some ‘timeless’ ethical questions, such as the risk of a quantum divide between the “haves” and “have-nots,” heightened privacy and security concerns, and the need to democratise the innovation process.

Recognising that the intensification of trends may be the most prominent ethical issue is as relevant for quantum technologies as it was for nanotechnology. Quantum technologies can be seen as ‘radical intensifiers’—technologies that enhance our existing capabilities to a significant degree. Roughly: sensors become more sensitive, computers faster, communication more secure. Certainly, by vastly improving certain capacities, quantum technologies enable actions that were previously (practically) impossible. However, this is better understood as radical intensification rather than a fundamental shift. Even the quantum cybersecurity threat, often framed as a new quantum-specific threat (National Institute of Standards and Technology, n.d.; QISS, n.d.), could be viewed as an extension of an ever-present issue, magnified by these enhanced capabilities.

One ‘timeless’ ethical issue that appears to be receiving less attention in the context of quantum technologies is the question of *desirability*. The general attitude towards quantum technologies seems to be overwhelmingly positive. This could be due to the fact that quantum technologies are relatively abstract to most people, lacking a concrete image of potential applications. This abstraction deprives the debate of a clear starting point for discussions about desirability. Addressing this challenge is critical: how can we engage in meaningful discussions about the desirability of quantum technologies in the absence of concrete visions of possible futures? This question leads us to the topic of (ir)responsible speculation, which we will explore next.

### (Ir)responsible Speculation?

The idea of a ‘speculation trap’ in NEST ethics was first highlighted during the debate on nanotechnology (Nordmann, 2007; Nordmann & Rip, 2009). At that time, the nano discourse faced criticism for its tendency to “foreshorten the future,” treating potential future scenarios as if they were immediate ethical issues warranting substantial reflection. In this respect, the quantum technology discourse bears more similarity to the nano debate than, for instance, the discussions surrounding AI. While part of the AI debate can be considered speculative—focusing on concepts like artificial general intelligence and the singularity (Walsch, 2017; Pilling & Coulton, 2019) - it is also grounded in actual applications and real-world incidents. In contrast, the quantum debate is almost entirely focused on potential futures, making it vulnerable to the same speculation trap.

As with nanotechnology, the prevailing attitude towards quantum technology is to “get it right from the outset.” This is reflected in efforts toward anticipatory governance (OECD, 2025) and, specifically, ‘upstream ethics’ (de Jong, 2025). However, engaging in ethics upstream is inherently challenged by a lack of knowledge and significant uncertainties about future developments. Thus, some degree of speculation seems unavoidable when engaging in ethics at such an early stage of technological development. The warnings against ‘irresponsible’ speculation that emerged during the nano debate do not yet seem to have been fully integrated into the quantum discourse. For instance, Roberson (2023) highlights a phenomenon of “quantum hype”, characterised by “overwhelming optimism for the field without tempering realism” (p. 5). The term “hype”, in the sense of speculative overreach, is particularly apt given that scalable prototypes for many of the promised quantum technologies remain largely absent. While Roberson acknowledges that hype can play a constructive role in raising awareness about a technology and its potential impacts, it can also have detrimental effects. Excessive hype may lead to disillusionment within the field, potentially resulting in a “quantum winter” (2023, p.2). Beyond the negative impact on funding and investment, quantum hype could also undermine the quality of the ethics debate (Nordmann, 2007).

If one key lesson from the nano discourse is that ethical reflection should involve a critical examination of technological promises—and a scrutiny of the very foundations and focus of the ethical discussion—then it is clear that we can and should enhance our approach to quantum ethics. This involves critically evaluating the quality of the promises made about quantum technologies. This call for scrutiny extends beyond individual promises to include a broader reflection on how future possibilities are envisioned and by whom. In other words, the quantum discourse would, especially at this stage of infancy, benefit from ethical reflection on the attribution of meaning in the emerging discourse.

Another lesson from the nano discourse, and a suggested remedy against irresponsible speculation, was the importance of distinguishing between a technology as a broad class and specific instances within that class. In the quantum discourse, this distinction is not consistently maintained. On one hand, quantum technologies are often described as a family of technologies, with three main branches: sensing, computation, and communication. So, the distinction is made in theory. However, discussions about the societal implications of quantum technologies tend to focus disproportionately on the implications of quantum computing in particular. This focus might be explained by (a) the perceived broader range of implications associated with quantum computing, compared to sensing, which has a more limited scope of application, and (b) the higher level of technological readiness in quantum computing compared to quantum communication, coupled with significant public and private investments in this field. However, if we were to base ethical reflection on the actual level of technological readiness, there would be more reason to focus on the ethical aspects of quantum sensing than is currently being done.

A third lesson concerning (ir)responsible speculation relates to the prevailing focus on potential consequences. Much like the discourse on nanotechnology, the conversation around quantum technologies appears to be dominated by a focus on potential impact (Swierstra & Rip, 2007). This emphasis on consequences can be problematic, as it often leads to speculative discussions, which are inherently uncertain and may detract from more grounded ethical considerations. To avoid falling into the speculation trap in quantum discourse by concentrating ethical reflection on imagined outcomes, we can learn from the nanoethics discourse that it is crucial to ‘stand in the present’ and to reflect on the discourse itself. As we have discussed, one way to do this is by reflecting on the visions about a future with quantum technologies, along with their proponents, and subject them to rigorous ethical scrutiny. As Urueña argues, vision assessment involves “identifying and considering which actors, knowledge, and assumptions have been included or excluded when establishing the arena of ‘the (im)plausible’ and/or ‘the (un)desirable’, and on what basis” (Urueña, 2021). This approach aligns with the growing need for an explicit discussion about the desirability of quantum technologies. Critically examining current visions could be a crucial first step in this direction. Given the technical complexity, high specialisation, and capital intensity of quantum technologies, this task is particularly urgent: if left unchallenged, powerful actors—scientists, corporations, and state entities—may come to define what is considered ethically relevant in ways that reinforce their own interests and positions. A critical, present-focused ethical approach should therefore address not only which futures are imagined, but also who gets to do the imagining.

### Ambivalence: Framing the Quantum Advantage

Ambivalence is also evident in quantum technology development. As described earlier, quantum technology is both new and not new, while methods ground in quantum mechanics can enable new applications. As mentioned previously, promises of a ‘quantum advantage’ (compared to the capabilities of classical devices) are to be found in specific applications. Indeed, much of the focus remains on the novel capabilities that quantum technologies are expected to unlock. Various actors, including scientists, corporate leaders, and state entities, emphasise the groundbreaking potential of quantum technologies, often amplifying the perceived novelty for strategic reasons. Conversely, more sceptical perspectives – those questioning whether quantum technologies will truly prove disruptive or even materialise as currently envisioned – are comparatively rare. As observed for the case of nanotechnology, we can expect ambivalence with respect to different actor strategies that may play out on the part of proponents and opponents of the new technology as priorities and paths of development become clear. This, in turn, will give substance to the form that quantum for good might take, at least in how it is understood and represented by certain constituencies.

## Conclusion

The embryonic stages of the development of quantum technologies offer an opening with which to shape ethics of and responsible innovation for quantum technology. This paper seeks to move away from the ‘novelty trap’ seen for previous engagements with NEST and related ethical discourse and to take stock of experiences and past achievements. It does so by adopting a ‘heuristic of continuity’, reflected in a focus on what we already know with respect to historical insights and the features of NEST. We mobilise the nanotechnology discourse and experience as a paradigmatic case of NEST-ethics discourse. We have pointed to a variety of features of nanoethics discourse, and used these as an analytic frame with which to spot continuities within the current quantum ethics discourse, drawing lessons from the nano debate to inform the quantum debate.

We emphasised that, like the nanotechnology debate, the ethical discourse surrounding quantum technology often highlights its novelty, potentially overlooking the fact that many ethical concerns are not entirely new but rather amplified versions of existing issues. We also identified ‘quantum for good’ as a variant of the responsible nanotechnology framework, arguing that it calls for a critical examination of its underlying normative aspirations. Additionally, we revisited the critique of speculative ethics from the nanoethics debate, which remains relevant to quantum technology, stressing the need to assess the quality of technological promises to maintain the relevance of the ethical discussion. Finally, we highlighted the ambivalence in both debates, which underscores the importance of recognising the active framing processes that shape ethical discourse.

Our emphasis on continuity may raise the concern that we understate the potential for rupture or radical discontinuities that quantum technologies might introduce. Our analysis acknowledges the unique and enabling features of quantum technologies, as well as their discontinuities when compared to nanotechnology. We do not deny that in cases of genuine rupture, revisiting—or even reinventing—ethical frameworks may be necessary. However, emphasising novelty risks obscuring important continuities in ethical discourse, which offer valuable opportunities to learn from past debates. We contend that, given its strong focus on what is new, current quantum discourse tends to overlook these opportunities. By framing ‘quantum ethics’ as a form of ‘NEST-ethics’, we seek to resist the lure of novelty and actively explore continuities with prior debates, aiming to contribute to a more reflective and historically informed ethics of quantum technologies.

From the outset, we highlighted our responsibility as ethicists and social scientists to resist being overly enticed by a focus on novelty of NEST. We have used the ‘grammar’ of NEST-ethics and offered key features of the nanotechnology discourse as heuristic tools with which to engage with the emerging ethics discourse around quantum technology development. At the same time, we have emphasised those moments in which ethics scholars are encouraged not only to confine themselves to questions and issues of continuity but to venture into more critical questioning and analysis of wider political developments - ethics must engage with the distribution of power, the political motivations behind technological development, and the actors who stand to benefit. By adding this ‘second order’ dimension to our ethical reflections, we assert that advancing the debate on responsible quantum technologies demands a deeper interrogation of politics, power, and social positioning.

Further research is needed to implement the principle of ‘standing in the present’ in the ethics discussion surrounding quantum technologies. As suggested, one way to achieve this is through the critical assessment of visions that shape future expectations of the technology. Additionally, empirical studies should investigate the power dynamics influencing the development of quantum technologies, examining which actors hold influence and how their interests shape both the technological landscape and its ethical discourse. This could also include exploring the geopolitical implications of quantum advancements.

In conclusion, this paper advocates for a grounded ethics discourse about quantum technologies, anchored in continuity and attentive to the broader societal and political contexts influencing technological development. Quantum technologies do not exist in a vacuum, and neither should our ethical discourse. The time to address these deeper issues is now, before the window of opportunity closes.

## Data Availability

Not applicable.

## Footnotes

1 Identifying an emphasis on novelty as a possible trap in ethical discussions inevitably raises the question of when ‘the usual’ transitions into something genuinely novel. We acknowledge that novelty is not an objective property but an interpretative assessment, and that it exists on a continuum rather than as a discrete category. Rather than attempting to define what constitutes novelty in absolute terms, we focus on how novelty is claimed and framed in discourse.

2
https://quantumdelta.nl/centre-for-quantum-and-society.

3 Quantum Impact on Societal Security (QISS) at the University of Amsterdam.

4 TA NanoNed was the Technology Assessment (TA) Program that was established in 2003 as part of the Dutch R&D consortium NanoNed (Nanotechnologie Nederland).

5 While the boundary between technology and science has always been inherently blurry, as they are often interdependent, our focus here is on highlighting that emerging technologies are still in the early stages of development. These technologies have not yet fully moved beyond the scientific phase—the phase of fundamental research—into more mature technological application. A more detailed discussion on the relationship between science and technology can be found in De Jong and De Haro (2025).

6 The Dutch Centre for Quantum and Society and Stanford Center for Responsible Quantum Technology.

7 The drivers for the two notions do differ, however; ‘tech for good’ has been positioned as emerging out of both public *and* private sectors, while ‘responsible development’ of nanotechnologies largely derived from particular public and institutional contexts in the European Union (EU) and the United States (US).

8 With “quantum ethics” we refer to ethics of quantum technology, similar to ’nanoethics‘referring to ethics of nano technology, and do not refer to the (potential) ethical implications of quantum theory.
